# The Impact of Cooking Classes on Food-Related Preferences, Attitudes, and Behaviors of School-Aged Children: A Systematic Review of the Evidence, 2003–2014

**DOI:** 10.5888/pcd11.140267

**Published:** 2014-11-06

**Authors:** Derek Hersch, Laura Perdue, Teresa Ambroz, Jackie L. Boucher

**Affiliations:** Author Affiliations: Laura Perdue, University of Minnesota Extension Regional Center, St. Cloud, Minnesota; Teresa Ambroz, Jackie L. Boucher, Minneapolis Heart Institute Foundation, Minneapolis, Minnesota.

## Abstract

**Introduction:**

Cooking programs have been used to promote healthful eating among people of all ages. This review assesses the evidence on childhood cooking programs and their association with changes in food-related preferences, attitudes, and behaviors of school-aged children.

**Methods:**

We systematically searched PubMed, Ovid-Medline, and CINAHL (Cumulative Index to Nursing and Allied Health Literature) databases. We included primary research articles that involved cooking education programs for children and searched reference lists for eligible articles. Studies considered for review contained a hands-on cooking intervention; had participants aged 5 to 12 years; were published in a peer-reviewed journal on or after January 1, 2003; and were written in English. We used the Effective Public Health Practice Project Quality Assessment Tool for Quantitative Studies to rate the strength of each article and assess bias. The following information was extracted from each study: study design, sample size, location, duration, intervention components, data collection methods, and outcomes.

**Results:**

Eight studies met the inclusion criteria and used cooking education to influence children’s food-related preferences, attitudes, and behaviors. Programs varied in duration, evaluation methods, and outcomes of interest. Self-reported food preparation skills, dietary intake, cooking confidence, fruit and vegetable preferences, attitudes toward food and cooking, and food-related knowledge were among the outcomes measured. Program exposure ranged from 2 sessions to regular instruction over 2 years, and the effect of cooking programs on children’s food-related preferences, attitudes, and behaviors varied among the reviewed studies.

**Conclusions:**

Findings suggest that cooking programs may positively influence children’s food-related preferences, attitudes, and behaviors. However, because study measurements varied widely, determining best practices was difficult. Further research is needed to fill knowledge gaps on ideal program length, long-term effects, and usefulness of parent engagement, tasting lessons, and other intervention components.

## Introduction

Since the 1980s, Americans have reduced the time they spend preparing and eating meals at home (1,2). The cause of this cultural shift is unknown, although several suggested factors are increased proportion of parents in the labor force, food accessibility, and time constraints from longer working hours (3–5). These factors — along with a lack of basic cooking skills, healthful eating knowledge, or both — may influence families and, in turn, children, to increase their consumption of foods away from home (2). This behavior is problematic, because restaurant meals often lack adequate amounts of fruits and vegetables and are often calorie-dense rather than nutrient-dense, which may result in poor diet quality and adverse health outcomes if such meals are consumed regularly (6–8).

Many interventions have attempted to increase consumption of and preferences for fruits and vegetables and influence other food-related preferences, attitudes, and behaviors among children (9). Although these efforts may be improving children’s overall health, it is unknown what intervention component is effective at prompting the desired changes (10). Prompted by the shift away from home food preparation, researchers have begun to study cooking programs as a way to positively affect participants’ food-related preferences, attitudes, and behaviors (5,11,12).

Although evidence suggests that cooking programs are effective at improving food-related preferences, attitudes, and behaviors among adolescents and adults, their effect on children remains uncertain (13–17). A previous review of this topic identified only 4 studies, published between 1995 and January 2008, and concluded that evidence on the benefits of cooking programs was lacking (18). Despite null findings, cooking programs have been recommended by public health professionals to address the obesity epidemic (3,5). This systematic review aims to assess the latest evidence concerning childhood cooking programs and their association with children’s food-related preferences, attitudes, and behaviors; inform future efforts; and identify gaps in the literature.

## Methods

### Data sources

This systematic review was conducted using methods developed by Thomas et al for public health research (19). Three databases, PubMed, Ovid-Medline, and CINAHL (Cumulative Index to Nursing and Allied Health Literature), were searched for primary research articles published between January 2003 and March 2014 that involved cooking education programs. This timeframe was chosen to obtain a sample of recent programs. An exhaustive search was conducted using medical subject headings and keywords including *cooking*, *education*, and *children*. A reference list search was also performed via articles that met the inclusion criteria and relevant papers in the field.

### Study selection

Studies were considered for review if they contained a cooking education intervention for children aged 5 to 12 years. This age range was selected on the basis of the average age of elementary school children in kindergarten through sixth grade; studies that included most children outside this age range were excluded. Interventions were the only studies of interest; therefore, randomized controlled trials (RCTs) and quasi-experimental studies were the only types of studies accepted. To adequately assess program impact, the minimum sample size allowed was 10. Cooking education programs were defined as those that occurred in a community or school setting and involved food preparation lessons. Studies were required to adequately describe the cooking intervention and at least include the frequency of cooking activities and types of recipes made. Those studies that only evaluated a previously conducted intervention or did not contain hands-on cooking by children were also excluded.

Study titles and abstracts retrieved from the initial database searches were independently screened by 2 authors to determine suitability for review. Author information and journal titles were not concealed from the reviewers. Articles with abstracts containing information that conflicted with at least 1 of the inclusion criteria were excluded. Full-text articles of abstracts meeting all of the inclusion criteria were obtained through the University of Minnesota library. Two review authors independently inspected each article to determine aptness for review inclusion. Final decisions for inclusion and exclusion were made by agreement among all listed authors (Figure).

**Figure:**
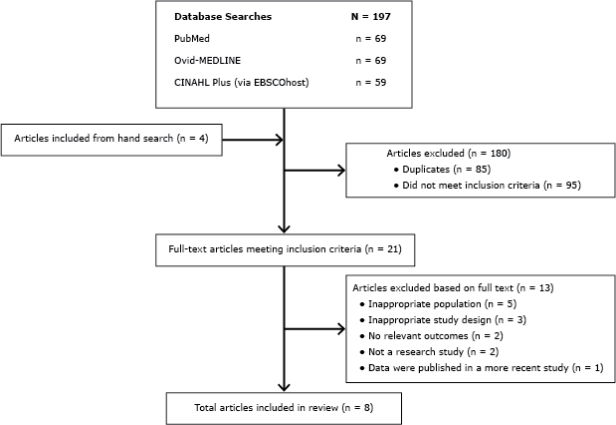
Flow diagram depicting systematic literature search of cooking education programs for children aged 5 to 12 years published between 2003 and 2014. Abbreviation: CINAHL, Cumulative Index to Nursing and Allied Health Literature.

### Data extraction

Study data were independently obtained by 2 authors who were not blind to the author information or journal titles. The following information was extracted from each study: study design, sample size, location, duration, intervention components, data collection methods, and outcomes. Study authors were not contacted, and only published information was extracted for this review. Quality assessments were independently conducted for each article by 2 reviewers. The Effective Public Health Practice Project (EPHPP) Quality Assessment Tool for Quantitative Studies was used to rate each article according to selection bias, study design, control of confounders, blinding, data collection methods, and withdrawal and drop-out rates (19). This assessment tool has been validated and recommended for use in systematic reviews of public health interventions to rate the methodological quality and validity of RCTs and quasi-experimental and uncontrolled studies (19–22). Study components were rated using the EPHPP tool as *strong*, *moderate*, or *weak*. Studies were rated strong overall if none of the components were rated weak, moderate overall if 1 component was rated weak, or weak overall if 2 or more components were rated weak. Because of the low number of published articles on this topic, articles rated as weak, moderate, or strong were reviewed. Institutional review board approval was not sought or required for this study, according to US Department of Health and Human Services guidelines (23).

## Results

### Overview of included studies

Eight articles met the inclusion criteria and were published between January 2003 and March 2014, in 7 different journals (Table 1) (24–31). Recruitment for all included studies occurred in the elementary or primary school setting; sample sizes ranged from 44 (29) to 1,230 (27). Six (24–27,30,31) of the interventions were conducted during the school day and integrated into the classroom curriculum, 1 (28) took place in an after-school program and was taught at a community garden, and the other study (29) occurred in the evening at a community center. Among 7 of the 8 studies, the median number of structured sessions was 10 (range, 2–12 sessions) (24–29,31). The final study, which was integrated into the participating schools’ curriculums, occurred on a weekly basis while school was in session for 2-and-a-half years, but the actual number of sessions was not reported (30). Duration of lessons were reported in 5 of the 8 studies, with a median time of 90 minutes (range, 90–120 minutes) (26–30). Three of the studies engaged parents, either through separate lessons (28,29) or a newsletter that was sent home (31).

**Table 1:** Characteristics of Included Studies that Involved Primary School-Aged Children and Contained a Cooking Component (n = 8)

Study Purpose	Study Design	EPHPP Rating	Sample Size	Duration	Intervention Components
**Caraher et al 2013 (** **24** **)**					
Determine the effectiveness of an in-school cooking program that uses chefs as instructors	2 group; quasi-experimental; pre–post assessment	Moderate	Intervention group: n = 86; control group: n = 83	2 sessions	Cooking lessons
**Cullen et al 2007 (** **25** **)**					
Increase fruit and vegetable consumption through a multimedia-based food preparation and eating behavior curriculum	Randomized; 1 group; pre–post assessment	Weak	Intervention group: n = 671	10 sessions over 5 weeks	Cooking lessons; nutrition education
**Cunningham-Sabo and Lohse 2013 (** **26** **)**					
Determine the impact of a cooking and tasting program on children’s cooking attitudes, cooking self-efficacy, and fruit and vegetable preferences	Randomized; 2 group; pre–post assessment	Strong	Intervention group: n = 137; control group: n = 120	3 two-hour cooking classes and 3 one-hour tasting sessions over 1 school semester	Cooking lessons; tasting activities
**Cunningham-Sabo and Lohse 2014 (** **27** **)**					
Compare the impact on children’s cooking attitudes, cooking self-efficacy, and fruit and vegetable preferences between a cooking and tasting program, a tasting-only program, and a control group	3 group; quasi-experimental; pre–post assessment	Strong	Cooking and tasting group: n = 539; tasting group: n = 294; control group: n = 397	5 two-hour cooking lessons and 5 one-hour tasting lessons during a 9-month school year	Cooking lessons; tasting activities
**Davis et al 2011 (** **28** **)**					
Determine the effects of a culturally focused, 12-week gardening and cooking program on dietary intake and health outcomes among predominantly Hispanic, fourth- and fifth-grade students	2 group; quasi-experimental; pre–post assessment	Moderate	Intervention group: n = 34; control group: n = 70	Twelve 45-minute nutrition and cooking lessons and twelve 45-minute gardening lessons over a 12-week period	Cooking lessons; nutrition education; gardening lessons
**Fulkerson et al 2010 (** **29** **)**					
Pilot a parent–child nutrition education program to increase family dinner frequency, parent self-efficacy in preparing healthy meals and child food preparation skills	2 group; experimental; post assessment	Moderate	Intervention group: n = 22; control group: n = 22	Five 90-minute sessions over a 10-week period	Cooking lessons; nutrition education; tasting activities; group meals
**Gibbs et al 2013 (** **30** **)**					
Determine the effectiveness of an in-school nutrition and gardening program on elementary school children’s willingness to try new foods	2 group; quasi-experimental; pre–post assessment	Weak	Intervention group: n = 463; control group: n = 280	Weekly 45-minute garden and 90-minute cooking classes, while school was in session, for 2.5 years	Cooking lessons; gardening lessons
**Quinn et al 2003 (** **31** **)**					
Improve attitudes toward and increase the fruit and vegetable consumption of fifth-grade students	2 group; quasi-experimental; pre–post assessment	Weak	Intervention group: n = 81; control group: n = 68	11 sessions	Cooking lessons; nutrition education

Abbreviation: EPHPP, Effective Public Health Practice Project.

The outcomes of interest and evaluation methods used varied (Table 2). Two interventions evaluated participants’ willingness to try new foods (30,31); 4 studies measured food preparation skills and cooking confidence (24,26,27,29). Among the 5 studies that conducted preintervention and postintervention dietary assessments, 2 used 24-hour dietary recalls (25,29), 1 used a food frequency questionnaire (28), 1 used both methods (31), and 1 (24) used self-reported consumption of selected vegetables. Fruit and vegetable preference was also measured in 3 studies using a qualitative scale (25–27). Anthropometrics were obtained in 2 of the interventions, with body mass index (BMI) as an outcome of interest (28,29); 1 study (28) also measured waist circumference, blood pressure, and total body fat.

**Table 2:** Outcomes of Interest, Evaluation Methods, and Major Findings of Included Studies (n = 8)

Outcome of Interest and Evaluation Method	Major Findings
**Caraher et al 2013 (** **24** **)**	
Cooking confidence, vegetable consumption, and confidence in asking for favorite vegetable assessed by child questionnaire	Increase in cooking confidence among the intervention and control groups; increase in vegetable consumption in the intervention group; confidence to ask parents for pasta salad ingredients increased in the intervention group
**Cullen et al 2007 (** **25** **)**	
Fruit and vegetable consumption assessed by 24-h dietary recall; fruit and vegetable preferences and self-efficacy for eating fruits and vegetables assessed by child questionnaire	An increase of 1 combined serving of fruit, 100% fruit juice, and vegetables was observed for participants who had the highest baseline consumption of fruits and vegetables and completed 2 or 3 goals; increase in vegetable consumption was observed among those with the highest baseline consumption that completed 0 preparation goals or 1 preparation goal
**Cunningham-Sabo and Lohse 2013 (** **26** **)**	
Fruit and vegetable preferences, attitudes toward cooking, and cooking self-efficacy assessed by child questionnaire	Participants in the treatment group had higher fruit preference scores, vegetable preference scores, and attitudes toward food and cooking and cooking self-efficacy than participants in the control group; baseline to follow-up changes were also greater in the treatment group than in the control group for vegetable preference scores, attitudes toward cooking, and food and cooking self-efficacy
**Cunningham-Sabo and Lohse 2014 (** **27** **)**	
Fruit and vegetable preferences, attitudes toward cooking, and cooking self-efficacy assessed by child questionnaire.	Participants in the cooking and tasting intervention had the highest increases in cooking self-efficacy; changes in fruit and vegetable preferences were greater among participants in the cooking and tasting group than among participants in the control group; changes in vegetable preferences were also greater among participants in both intervention groups than among those in the control groups
**Davis et al 2011 (** **28** **)**	
Overall health measured by BMI, total body fat, waist circumference, and blood pressure; dietary intake assessed by 41-item food frequency questionnaire	Dietary fiber intake increased by 22% among participants in the intervention group, and dietary fiber intake decreased by 12% among participants in the control group; diastolic blood pressure decreased more among participants in the intervention group than among those in the control group; overweight participants in the intervention group gained less weight and had a greater improvement in BMI than overweight participants in the control group
**Fulkerson et al 2010 (** **29** **)**	
Frequency of family dinners, food sources, parental self-efficacy regarding healthful changes at home and child’s food preparation skill assessed by parent questionnaire; food preparation skills assessed by child questionnaire; obesity status measured by BMI; home food availability assessed by home food inventory tool; family meal quality assessed by brief mealtime screener tool; dietary intake assessed by 24-hour recall	Children in the intervention group rated their food preparation skills higher than did participants in the control group; by parent report, child participation in meal preparation was higher in the intervention group than it was among children in the control group
**Gibbs et al 2013 (** **30** **)**	
Willingness to try new foods assessed by parent and child questionnaires; food choices and ability to describe foods assessed by child questionnaire	Children’s willingness to try a new food if they had never tried it, cooked it, or grown it increased more among participants in the intervention schools than among participants in the control schools
**Quinn et al 2003 (** **31** **)**	
Dietary intake assessed by 7-item fruit and vegetable food frequency questionnaire and 24-hour dietary recall; food-related knowledge, attitudes toward food, willingness to try new vegetables, exposure to healthful foods, and eating habits assessed by child questionnaire; perception of children’s attitudes and eating habits and household cooking and purchasing habits assessed by parent questionnaire	Participants in the intervention group consumed more fiber than did participants in the control group; participants in the intervention group increased dietary folate, fruit servings, and milk servings; students in the intervention group were more willing to try new vegetables than were children in the control group; 44% of parents reported an increase in the amount of fruit and vegetables their children were eating since the program was completed

Abbreviation: BMI, body mass index.

Using the EPHPP tool (19), 2 (26,27) articles were considered strong in quality, 3 (24,28,29) moderate in quality, and 3 (25,30,31) weak in quality (Table 1). The selection bias and confounding components of the assessment resulted in the most *weak* ratings, with 3 studies each. Data collection methods and withdrawals and drop-outs had the most *strong* ratings, with 5 studies qualifying. Few similarities existed between studies’ quality ratings, except for Caraher et al (24)and Davis et al (28), which had identical ratings.

### Effects of interventions

Outcomes of the included studies are summarized in Table 2. The 2 studies that assessed children’s willingness to try new foods found an increase postintervention in the intervention group compared with baseline (30) or a control group (31). Children were also more willing to try new foods if they had cooked or had grown it (30). When parents were asked about their child’s willingness to always try new foods, a nonsignificant increase was observed (30). Caraher et al qualitatively measured students’ preference for the 5 vegetables used in the class recipe (24). No significant change was observed in the control group’s responses, but a significant increase was observed among those in the intervention group. Attitudes toward cooking and food were measured by self-report questionnaires in both studies conducted by Cunningham-Sabo and Lohse (26,27). Among fourth-graders in Colorado, changes in attitudes toward cooking and food were significantly greater among the intervention group than among the control group (26). Among children in New Mexico, cooking attitudes did not significantly change, regardless of the intervention group (27).

Food preparation skills and cooking confidence were determined in 2 studies on the basis of participants’ reported ability to cut up fruits and vegetables, follow a recipe, and measure ingredients, among several other food preparation actions (24,29). Caraher et al observed a significant increase in cooking confidence scores (on a scale from 1 to 4) among both the intervention and control groups from baseline to follow-up (24). Fulkerson et al compared the food preparation skills of children and parents in intervention and control groups; they found a significant difference among the children but not among the parents (29). Cunningham-Sabo et al also measured cooking self-efficacy and found significant improvements among fourth graders in Colorado (26) and New Mexico (27).

Among the 4 studies that measured children’s preintervention and postintervention daily servings of fruits and vegetables, 1 (25) found significant increases from baseline in both fruit and vegetable consumption and 1 (31) found a significant increase from baseline for fruit consumption only. Nonsignificant increases from baseline were observed in the 2 other studies (28,29). Caraher et al (24) used self-reported consumption of selected vegetables as a proxy for overall vegetable consumption. Participants were asked preintervention and postintervention if they had consumed any of the 5 vegetables at least once over the past week. Although no difference was observed in the control group at follow-up, the proportion who reported eating 1 or more of the vegetables in the intervention group significantly increased from baseline. Two studies (26,27) out of 3 that measured children’s preferences for fruits, vegetables, or both found a significant increase from preintervention to postintervention; the third did not compare preintervention with postintervention and used the comparison only as an adjustor in the analysis (25).

Among the studies that measured physical characteristics, findings were mixed. Two interventions (28,29) found nonsignificant changes in BMI from baseline to follow-up, and 1 (28) found no change in total body fat percentage from baseline to follow-up. However, Davis et al observed a significant decrease in BMI from baseline to follow-up among overweight and obese participants in the intervention group compared with overweight and obese participants in the control group (28). Improvements in diastolic blood pressure from baseline to follow-up were significantly different between treatment and control groups (28).

### Study quality

Overall study quality, as measured by the EPHPP tool (19), of the included studies ranged from weak to strong. Three studies (29–31) likely are subject to selection bias; samples were selected for convenience or participation was self-selected. Regarding study design, 2 (26,29) were RCTs, 4 (24,27,28,31) were quasi-experimental with control groups, and 1 (25) randomly selected individuals for the intervention but did not have a control group. Three studies (24,25,28) lacked appropriate consideration for confounders. Blinding was not mentioned in any of the included studies, and whether participants’ responses were influenced by knowledge of the research aims is unclear. Data collection methods and tools were reported to be valid and reliable in 4 studies (24,26–28), reliable in 1 (29), with no information given in the remaining 2 (30,31). Five studies (24,26–29) reported participation rates greater than 80%, 2 (30,31) had rates between 60% and 79%, and 1 (25) did not report participation rates.

## Discussion

Given the rise in childhood obesity and known cultural shifts away from cooking, a review of cooking programs targeting elementary school children was conducted to understand program design and outcomes and to inform research gaps. Similar to findings of previous systematic and informal reviews of the literature, we found limited scientific articles written about the effectiveness of cooking interventions on children’s food-related preferences, attitudes, and behaviors (5,18).

Analyzing studies with intervention lengths ranging from 2 sessions to regular instruction over 2 years and with diverse outcome measurements makes determining best practices difficult. Data collection methods also differed greatly among studies; only 2 took physical measurements (28,29), and fruit and vegetable consumption was estimated predominantly by self-report or parent report. Because these collection methods vary in their reliability, generalizing the reviewed programs’ effectiveness at influencing food-related preferences, attitudes, and behaviors is challenging. The availability of tools to effectively measure behavioral and dietary characteristics, especially among children, is a limitation in the field of nutrition (32,33).

Given that only 2 (26,27) of the studies reviewed were considered strong according to EPHPP criteria, there appears to be a lack of high-quality intervention studies on childhood cooking programs. The literature lacks an adequate number of studies that effectively randomize participants to treatment and control groups. In most cases, this lack of randomization results from the availability of schools that are willing and able to accommodate a cooking program. Inadequate funding may also be an issue, given that randomization of schools and stronger study designs also require significantly more resources. Strict curriculum requirements may also affect study design and researchers’ ability to implement cooking interventions in schools; many schools do not have the time to include additional lessons, resulting in a small pool of possible schools and, in turn, participants who can be adequately randomized. As a result, researchers may not have been able to obtain a representative sample.

Despite various differences in delivery, each program had a significant effect on 1 or more of its participants’ food-related preferences, attitudes, and behaviors, although this finding could be attributed to publication bias. In studies that measured it, children’s willingness to try fruits and vegetables significantly increased after the cooking intervention (24,30,31). Furthermore, participants’ fruit and vegetable consumption, as reported by their parents, also significantly increased (24,31). In one case, these improvements were observed after only 2 cooking lessons (24). Although these short-term improvements are promising from a feasibility standpoint, repeated exposures are suggested to increase children’s preference for fruits and vegetables (34–36). Longer programs can incorporate more cooking skills, provide in-depth nutrition education, and better incorporate a culture of wellness into the school, the community, or both. It is also unclear whether participants benefited from having their parents involved in the cooking classes. None of the studies measured the impact that the programs had on parents’ food-related preferences, attitudes, and behaviors, although cooking programs for adults have had a modest impact on diet quality and food preferences (17).

Although some programs had a significant short-term impact on children’s food-related preferences, attitudes, and behaviors, the long-term sustainability of these changes is unknown. One study conducted follow-up surveys 6 months postintervention, but the results were not published (29). As more evidence suggests that childhood weight status is a good predictor of adult weight status, long-term evaluations that measure sustainability are needed to identify effective intervention strategies. Given that food-related preferences, attitudes, and behaviors can change dramatically throughout grade school, teaching sustainable healthful habits at a young age could have dramatic implications (37). However, the literature lacks substantial evidence about whether healthful habits taught at a young age are maintained.

This systematic review has some limitations. Efforts were made to capture all available published studies related to the aim of the review. However, selection and publication bias, inherent to the research modality, may be present. Also, if articles did not fully describe the cooking interventions, they were excluded without contacting authors; therefore, an article could have met the inclusion criteria if more information had been provided.

Our findings indicate that cooking education programs may be a promising tool for promoting positive changes in children’s food-related preferences, attitudes, and behaviors. Although no best practices or consistent themes were found among the successful interventions, gaps in the evidence were identified to inform future studies. What components are necessary for an effective program is unclear. Several design elements of cooking programs also require further research: where programs should occur, the ideal number of exposures, whether nutrition education should be paired with cooking lessons, the role of parent involvement, and the usefulness of tastings and gardening activities. In terms of program outcomes, more evidence is needed to determine whether changes in food choices are occurring as a result of cooking interventions and whether changes are sustained through childhood and adolescence. Future studies should address these gaps through controlled trials that measure both quantitative and qualitative effects; ideally they should be conducted in various environments such as schools, community centers, and the home.
